# Characteristics of the mitochondrial genome of Qinling zokor (*Eospalax rufescens*)

**DOI:** 10.1080/23802359.2020.1768919

**Published:** 2020-05-27

**Authors:** Pengfei Song, Hongmei Gao, Feng Jiang, Tongzuo Zhang, Zhenyuan Cai

**Affiliations:** a Key Laboratory of Adaptation and Evolution of Plateau Biota, Northwest Institute of Plateau Biology, Chinese Academy of Sciences, Xining, China; b Qinghai Provincial Key Laboratory of Animal Ecological Genomics, Xining, China; c College of Life Sciences, University of Chinese Academy of Sciences, Beijing, China

**Keywords:** Qinling zokor, *Eospalax rufescens*, mitochondrial genome, next generation sequencing

## Abstract

We report the complete mitochondrial genome for the *Eospalax rufescens*, a typical subterranean rodent species and endemic in China. The resulting *E. rufescens* mitogenome is 16,355 bases in size, containing 13 protein-coding genes (PCGs), 2 ribosomal RNA (rRNA) genes, 22 transfer RNA (tRNA) genes, and 1 noncoding control region (D-loop). The base compositions present highly biased toward A + T nucleotides. Moreover, twelve of all 13 PCGs initiated with ATN start codon, whereas *ND1* began with GTG start codon. Stop codons in 13 PCGs were all typical types except incomplete stop codon T for *ND4*. We further provide a Maximum Likelihood phylogenetic tree showing relationships among *E. rufescens* and other common subterranean rodents in family Spalacidae.

The Qinling zokor, *Eospalax rufescens*, is a typical subterranean rodent species and endemic in China (Wang 2003) belonging to subfamily Myospalacinae, family Spalacidae (Norris et al. 2004). Here, we provide the first complete mitogenome of *E. rufescens* (GenBank accession: MH933740).

The samples of *E. rufescens* was collected from Shaanxi province of China (N34.13°, E107.15°, altitude 1783 m) and currently stored at Key Laboratory of Adaptation and Evolution of Plateau Biota, Northwest Institute of Plateau Biology, Chinese Academy of Sciences under voucher number TBX-03. Total genomic DNA was extracted from muscle using DNeasy Tissue Kit (QIAGEN). An Illumina library was generated from genomic DNA and sequenced on an Illumina Hiseq 2500 platform. The mitochondrial genomes were reconstructed by MitoZ (Meng et al. 2019) with the default parameters, and the complete mitogenome sequence of *Myospalax aspalax* (GenBank accession number: KP724691) was used as a reference. The Maximum Likelihood phylogenetic tree was conducted with RA × ML v8.2.12 (Stamatakis 2014) through the online CIPRES Science gateway (Miller et al. 2010).

A total of 35,172,146 sequence reads were generated by next-generation sequencing after removing adaptor polluted reads, more than 5% Ns reads and low-quality sequences. The accurate annotated mitochondrial genome sequence of *E. rufescens* is a circular double-strand DNA molecule of 16,350 bp, containing 13 protein-coding genes (PCGs), 2 ribosomal RNA genes (rRNAs), 22 transfer RNA genes (tRNAs), and 1 non-coding region. The arrangement of the multiple genes is similar in line with other Myospalacinae species (Liu et al. 2011; Su et al. 2013; Li et al. 2016; Yuan et al. 2016; Cai et al. 2019). The overall base composition of the heavy strand is 32.81% A, 23.80% C, 31.12% T, and 12.96% G. The base compositions present highly biased toward A + T nucleotides. The heavy strand (H-strand) encodes 2 rRNA genes, 12 PCGs and 14 tRNA genes, the nicotinamide adenine dinucleotide dehydrogenase subunit 6 (*ND6*) gene and 8 other tRNA genes are encoded on the L-strand.

Nine of all 13 PCGs initiated with an ATG start codon except for *ND1*, *ND2*, *ND3*, and *ND5*, which began with GTG, ATT, ATA, and ATT start codons, respectively. Eight of the 13 PCGs use TAA as stop codon. The *ND1*, *ND2*, *CO1* and *ND6* are stopped with TAG, and *ND4* end with incomplete stop codon T. The tRNA genes range from 60 to 75 bp in length and employ the anticodons typical of vertebrate mt-tRNAs. All the tRNAs had a typical secondary structure (cloverleaf structure) except the *tRNA-Ser* (GCT), whose complete dihydrouridine arm was lacking. The 12S and 16S ribosomal RNA genes are 939 and 1571 bp long, respectively. In our study, the noncoding control region of the *E. rufescens* mtDNA is 937 bp long.

The results of phylogenetic analysis displayed that *E. rufescens* formed a clade with other species in subfamily Myospalacinae, and Rhizomyinae was a basal clade relative to others within Spalacidae (Figure 1). This mitogenome sequence of *E. rufescens* would help in evolutionary biology study of the Mysopalacinae.

**Figure 1.**
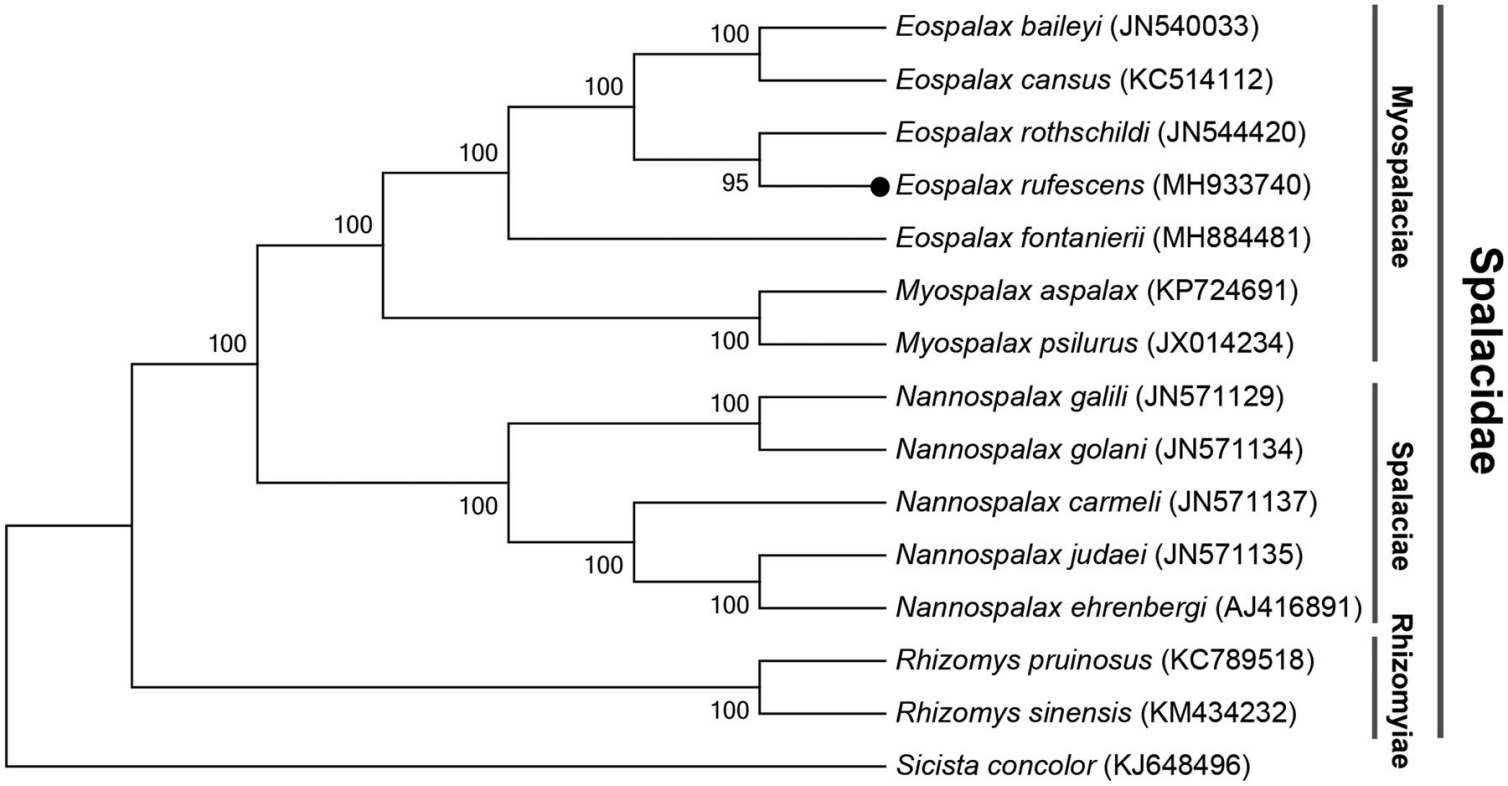
Maximum-likelihood (ML) phylogenetic tree of *Eospalax rufescens* and the other 12 species of Spalacidae using *Sicista concolor* as an outgroup. The number around each node indicates the ML bootstrap support values.

## Data Availability

The data that support the findings of this study are openly available in NCBI Sequence Read Archive (SRA) at https://trace.ncbi.nlm.nih.gov/Traces/sra/sra.cgi?view=search_obj, reference number SRR11637900.
